# Draft Genome Sequence of *Lactobacillus sakei* Strain FBL1, a Probiotic Bacterium Isolated from *Mukeunji*, a Long-Fermented Kimchi, in South Korea

**DOI:** 10.1128/genomeA.00365-16

**Published:** 2016-05-12

**Authors:** Jae-Hwan Kim, Eiseul Kim, Chang-Gyeom Kim, Dong-Won Choo, Hae-Yeong Kim

**Affiliations:** a Institute of Life Sciences and Resources, Graduate School of Biotechnology, Kyung Hee University, Yongin, Republic of Korea; b Department of Bioinformatics and Biosystems, Korea Polytechnics, Seongnam, Republic of Korea

## Abstract

This report describes the 2,032,158-bp draft genome sequence of *Lactobacillus sakei* (*L. sakei*) strain FBL1, isolated from *mukeunji* purchased at the Gwangju World Kimchi Culture Festival in 2012, South Korea. The total draft genome size was 2,032,158 bp with a G+C content of 41.2%.

## GENOME ANNOUNCEMENT

*Lactobacillus sakei* (*L. sakei*) is a facultative heterofermentative and Gram-positive anaerobic lactic acid bacterium (LAB). This bacterium, originally described in rice alcohol or sake, is commonly found in fresh meat and fish and is used as a starter culture (1). The complete genome sequence of *L. sakei* 23K (GenBank accession no. CR936503.1) was reported in 2005 (2). It consists of the circular chromosome containing 1,884,661 bp and 1,883 protein coding genes.

The genome sequence of *L. sakei* strain FBL1 with bile acid tolerance, isolated from *mukeunji*, a long-fermented kimchi (3), was analyzed in this study. *L. sakei* FBL1 was isolated from commercial *mukeunji* and showed 99% identity with the *L. sakei* strain DGH5 (KF469177.2) 16S rRNA gene. Comparative genomic analysis revealed 84% similarity with *L. sakei* 23K.

Genomic DNA was extracted with the G-spin genomic extraction kit (Intron Biotechnology Co., Republic of Korea) and quality was evaluated by the Agilent 2100 bioanalyzer with the high-sensitivity DNA kit. Draft genome sequencing was performed using an Ion Torrent Personal Genome Machine (PGM) system (Life Technologies, Germany) and a 318 semiconductor chip with 400-bp sequencing reads. A total of 4,048,495 reads, with an average read length of 320 bp, were generated. Reference-based assembly was carried out using SPAdes version 3.1.0, with *L. sakei* 23K as the reference genome. Assembly of the reads resulted in 44 contigs (≥500 bp, 541 to 287,097 bp, *N*_50_ length 116,042 bp). The total draft genome size was 2,005,160 bp with a G+C content of 41.0%. A total of 1,972 coding sequences, 76 RNA genes, and 296 subsystems were revealed by the RAST server (4, 5). The genome includes the production of a colicin V synthesis protein, adhesion and multidrug resistance efflux pumps, as well as those containing resistance genes, beta-lactam antibiotics, bile salt (choloylglycine hydrolase), and fluoroquinolones. Compared to the *L. sakei* 23-K genome, about 32 genes are missing. Importantly, phage proteins, choline binding protein, beta-galactosidase, phosphotransferase systems, and cobalt-zinc-cadmium resistance protein existed in *L. sakei* strain FBL1.

### Nucleotide sequence accession numbers.

This whole-genome shotgun project has been deposited in DDBJ/ENA/GenBank under the accession number LSFF00000000. The version described in this paper is the first version, LSFF01000000.

## References

[1] Najjari A, Ouzari H, Boudabous A, Zagorec M 2008 Method for reliable isolation of *Lactobacillus sakei* strains originating from Tunisian seafood and meat products. Int J Food Microbiol 121:342–351. doi:10.1016/j.ijfoodmicro.2007.11.045. 18155310

[2] Cornet M, Crutz-Le Coq AM, Dudez AM, Martin V, Beaufils S, Darbon-Rongère E, Bossy R, Loux V, Zagorec M 2005 The complete genome sequence of the meat-borne lactic acid bacterium *Lactobacillus sakei* 23K. Nat Biotechnol 23:1527–1533. doi:10.1038/nbt1160. 16273110

[3] Kim JH, Li J, Han SK, Qin P, Kim J, Park Y, Lee SY, Hong Y, Kim W, Kim HY 2016 Characterization of macrophage-activating lactic acid bacteria isolated form *mukeunji*. Food Sci Biotechnol 25:595–599. doi:10.1007/s10068-016-0083-x. PMC6049194 30263311

[4] Aziz RK, Bartels D, Best AA, DeJongh M, Disz T, Edwards RA, Formsma K, Gerdes S, Glass EM, Kubal M, Meyer F, Olsen GJ, Olson R, Osterman AL, Overbeek RA, McNeil LK, Paarmann D, Paczian T, Parrello B, Pusch GD, Reich C, Stevens R, Vassieva O, Vonstein V, Wilke A, Zagnitko O 2008 The RAST server: Rapid Annotations using Subsystems Technology. BMC Genomics 9:75. doi:10.1186/1471-2164-9-75. 18261238 PMC2265698

[5] Overbeek R, Olson R, Pusch GD, Olsen GJ, Davis JJ, Disz T, Edwards RA, Gerdes S, Parrello B, Shukla M, Vonstein V, Wattam AR, Xia F, Stevens R 2014 The SEED and the Rapid Annotation of microbial genomes using Subsystems Technology (RAST). Nucleic Acids Res 42:D206–D214. doi:10.1093/nar/gkt1226. 24293654 PMC3965101

